# Sex differences in outcomes after stroke among patients with low total cholesterol levels: a large hospital-based prospective study

**DOI:** 10.1186/s13293-016-0109-3

**Published:** 2016-11-24

**Authors:** Guanen Zhou, Zhongping An, Wenjuan Zhao, Yan Hong, Haolin Xin, Xianjia Ning, Jinghua Wang

**Affiliations:** 1 Department of Neurology, Tianjin Huanhu Hospital, 6 Jizhao Road, Jinnan District, Tianjin 300350 China; 2 Tianjin Key Laboratory of Cerebral Vascular and Neurodegenerative Disease, Tianjin, 300350 China; 3 Department of Epidemiology, Tianjin Neurological Institute, Tianjin, 300052 China; 4 Department of Neurology, Tianjin Medical University General Hospital, Tianjin, 300052 China

**Keywords:** Total cholesterol, Ischemic stroke, Outcomes, Sex differences

## Abstract

**Background:**

Previous studies have shown that total cholesterol (TC) levels are associated with stroke outcomes, but sex differences in the association between TC levels, especially a low TC level, and ischemic stroke outcomes are unknown. We aimed to assess the sex differences in stroke outcomes among patients with atherothrombotic infarctions and low TC levels in China.

**Methods:**

This study recruited patients with atherothrombotic infarctions from Tianjin, China, between May 2005 and September 2014. Patients with low TC levels (defined as TC <4.22 mmol/L) were analyzed in this study. Sex differences in stroke subtypes, severity, risk factors, and outcomes at 3 and 12 months after stroke were compared.

**Results:**

Overall, 1587 patients with low TC levels were recruited to this study from among 6407 patients with atherothrombotic infarctions listed in a stroke registry. Women were more likely than men to have posterior circulation infarcts, severe stroke, hypertension, and obesity but less likely to be current smokers or to consume alcohol. There were no sex differences in stroke outcomes. Older age and severe stroke were common risk factors for poor outcomes after stroke in this study. The presence of diabetes mellitus was an independent predictor of low mortality at 12 months after stroke, possibly because a drug commonly used to treat diabetes, metformin, enhances angiogenesis. Obesity was the determinant of the recurrence and dependency rates at 12 months after stroke.

**Conclusions:**

These findings suggest that patients (both men and women) with atherothrombotic infarction who have low TC levels would not benefit from receiving statin treatment. Therefore, it is crucial to explore the impact of statin treatment on outcomes in Asian patients, especially Chinese patients with atherothrombotic and low TC levels, in order to improve outcomes after stroke and reduce the disease burden.

## Background

Although age-standardized rates of stroke mortality have decreased worldwide in the past few decades, the global burden of stroke disability-adjusted life-years is a crucial health issue due to the increasing absolute number of stroke survivors [1, 2]. In 2014, in China, stroke was the third most common cause of death overall, the third in urban areas, and the second in rural areas [3]. Moreover, stroke is also a leading cause of functional impairments, with 20% of survivors requiring institutional care after 3 months and 15–30% being permanently disabled [4].

High total cholesterol (TC) level is a well-documented risk factor for coronary disease [5, 6]. Moreover, hypercholesterolemia has been well-documented as a modifiable risk factor for ischemic stroke [7], although a lower TC level was shown to be an independent predictor of hemorrhagic stroke in previous studies [8, 9]. The association between TC levels and stroke outcomes is controversial. A large number of studies have indicated that high TC levels were associated with better stroke outcomes [7, 10, 11], but the reverse trend was observed in other studies [12, 13].

With recent economic development, the incidence of stroke in China has increased dramatically [14]; however, large-scale studies of the association between TC level and stroke outcomes in China are rare, especially in patients with atherothrombotic infarction.

Therefore, we aimed to assess the sex differences in the associations between low TC levels on admission and long-term stroke outcomes after acute ischemic stroke (AIS) in patients with atherothrombotic infarction in China.

## Methods

### Patients

This was a hospital-based follow-up study using the Stroke Registry System that we developed in 2005 in Tianjin Huanhu Hospital, a specialized neurological hospital in Tianjin, China. All consecutive patients with first-ever AIS who were admitted to the Stroke Unit at Tianjin Huanhu Hospital within 72 h of stroke onset between May 2005 and September 2014 were recruited to this study. A clinical diagnosis of stroke was made according to the World Health Organization criteria and confirmed by neuroimaging (computed tomography/magnetic resonance imaging) [15]. Cases of transient ischemic attack were excluded from this study. The study originally included all patients with atherothrombotic infarction classified according to the Trial of Org 10172 in Acute Stroke Treatment (TOAST) for large-artery atherothrombosis and small-artery occlusion (SAO) [16], who were treated using statins and for whom data on TC level on admission were available. The patients were further categorized into two groups according to TC level on admission: the low-TC group, defined as patients with a TC level <4.22 mmol/L, and the non-low-TC group, defined as patients with a TC level ≥4.22 mmol/L. For this study, the final study population included only patients with low TC.

### Ethics, consent, and permissions

The study was approved by the Ethics Committee for Medical Research at Tianjin Huanhu Hospital, and written informed consent was obtained from each participant during recruitment.

### Data collection and group assignments

Detailed information on ischemic stroke subtype, stroke severity, previous history of diseases, stroke risk factors, laboratory examination results, and outcomes at 3 and 12 months after stroke were obtained from a standardized questionnaire and recorded in the Stroke Registry System.

To ensure data quality, three groups of senior trained neurologists (the assessment group, the follow-up group, and the quality control group) were responsible for determining the nervous system score at admission, for the reexamination (including of neurological score, risk factor management, and directing the treatment and rehabilitation) during follow-up, and for a sampled confirmation of 20% of all patients each month, respectively.

### Stroke subtypes

Stroke subtypes were defined as total anterior circulation infarct (TACI), partial anterior circulation infarct (PACI), posterior circulation infarct (POCI), and lacunar infarct (LACI) according to the Oxfordshire Community Stroke Project (OCSP) classification criteria [17].

### Neurological function deficits and stroke severity

Neurological function deficits were defined using the National Institutes of Health stroke scale (NIHSS), Barthel index (BI) [18], and modified Rankin scale (mRS) on admission [19]. Stroke severity was categorized into three groups on the basis of the NIHSS score: mild (NIHSS ≤7), moderate (NIHSS between 8 and 16), and severe (NIHSS ≥17) [20].

### Risk factors

Stroke risk factors included a medical history of hypertension (defined as a self-reported history of hypertension or the use of antihypertension drugs), diabetes mellitus (DM, defined as a history of DM or the use of hypoglycemic medications at discharge), atrial fibrillation (AF, defined as a history of AF, confirmed by at least one electrocardiogram, or the presence of arrhythmia during hospitalization), and obesity (body mass index ≥30 kg/m^2^) and modifiable lifestyle factors, including current smoking status and alcohol consumption.

### Definitions of outcomes

Stroke outcomes were described on the basis of mortality, recurrence, and dependency rates at 3 and 12 months after stroke. Outcomes were assessed using face-to-face or telephone follow-up interviews. Death was defined as all-cause cumulative death at the corresponding follow-up time points after stroke, and this information was collected from medical records or patients’ family members by telephone follow-up. Recurrence was defined as new-onset vascular events (stroke, myocardial infarction, and venous thrombosis) 30 days after the initial stroke in all survivors who completed follow-up using face-to-face interviews or telephone calls. Dependency was defined as an mRS score >2 among all survivors who underwent follow-up using face-to-face interview or telephone calls [21].

### Follow-up period

Follow-up was conducted according to a predetermined procedure. Trained neurologists reexamined patients in the outpatient department at 3 and 12 months after stroke. All patients completed follow-up by face-to-face interview, except for patients who were reexamined in their neighboring hospitals; these patients completed follow-up by telephone.

### Statistical analysis

Age is presented as mean (standard deviation), and NIHSS, BI, and mRS scores are presented as medians (interquartile ranges). These continuous variables were compared between men and women using the Student *t* test or the Mann-Whitney *U* test, as appropriate. At the different follow-up time periods after stroke, categorical variables, including stroke subtype, stroke severity, risk factors, and outcomes, are presented as number (percentage), and the trends were compared using chi-squared tests. Associations between the relevant risk factors and outcomes in men and women were assessed individually using univariate and multivariate logistic regression models and are presented as unadjusted and adjusted (by age, stroke severity, stroke subtypes, and risk factors) odds ratios (ORs), respectively, with 95% confidence intervals (CIs). All statistical analyses were performed using SPSS version 15.0 (SPSS Inc., Chicago, IL), and two-tailed *P* values <0.05 were considered statistically significant.

## Results

### Patient selection

**Fig. 1:**
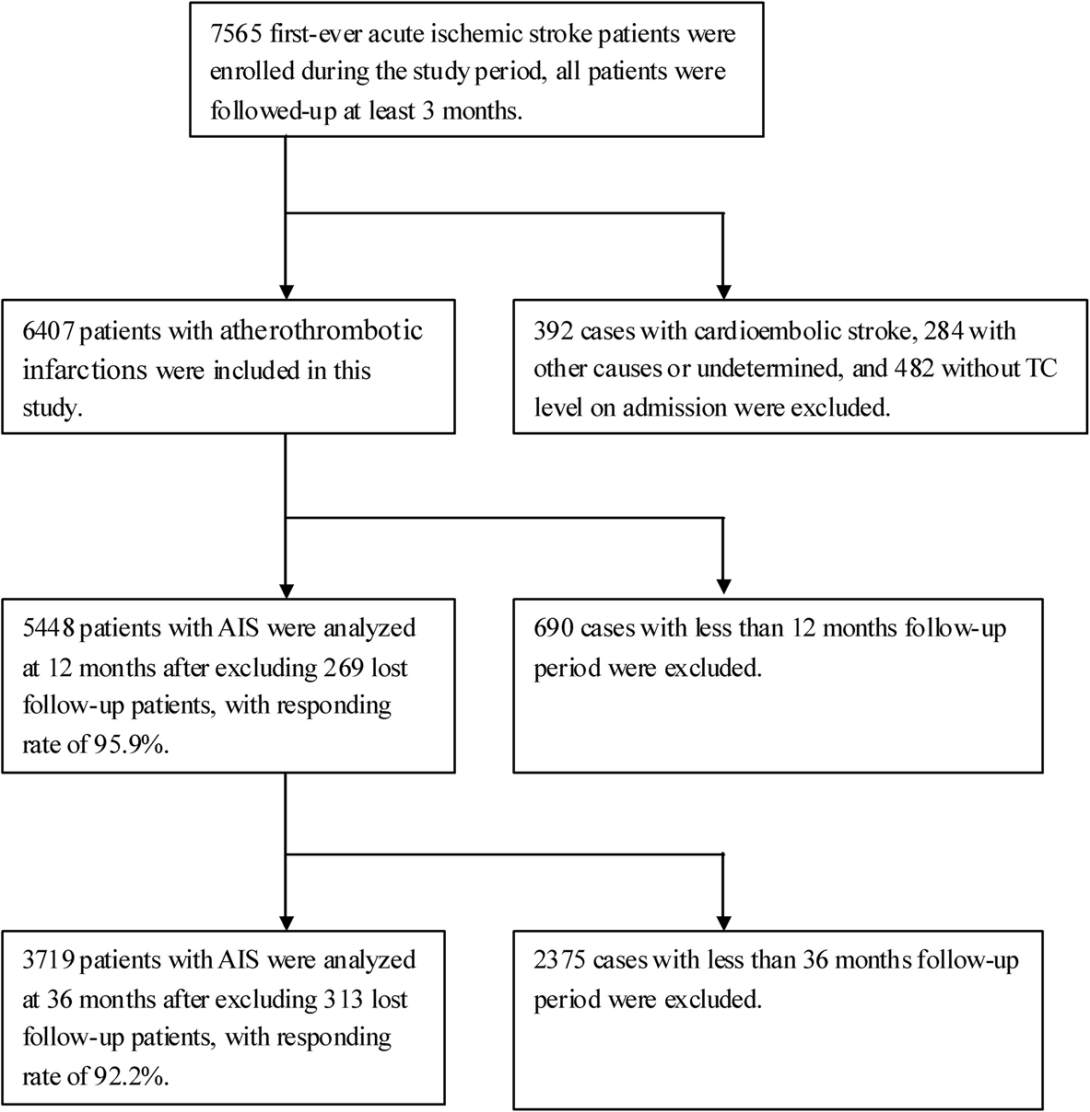
Flow diagram of participants

Of the 7565 AIS patients recruited between May 2005 and September 2014, 392 patients with cardioembolic stroke, 284 patients with other stroke and stroke of undetermined causes, and 482 patients without a TC level recorded on admission were excluded, resulting in 6407 patients with atherothrombotic infarction that were included. Of these, there were 1587 patients with low TC levels. After excluding those patients who did not complete follow-up, the response rate was 97.4% at 3 months and 94.9% at 12 months (Fig. 1).

### Sex differences in clinical features among patients with atherothrombotic infarction

Of the 1578 patients with low TC levels, 1272 (80.2%) were men and 315 (19.8%) were women. Women were more likely than men to have PACI (33.0 vs. 31.5%, *P* = 0.006), hypertension (80.6 vs. 71.4%, *P* < 0.001), and obesity (18.1 vs. 7.9%, *P* < 0.001), but men were more likely than women to have mild stroke (69.9 vs. 64.8%, *P* < 0.001), to be current smokers (49.4 vs. 13.0%, *P* < 0.001), or to drink alcohol (26.3 vs. 1.3%, *P* < 0.001). Moreover, women showed poorer neurological function (Table 1).

**Table 1:** Sex differences in clinical characteristics and risk factors among patients with low TC level and atherosclerotic stroke

Characteristics	Total	Men	Women	*P*
Cases, *n* (%)	1587	1272 (29.5)	315 (15.1)	–
Age, year, mean (SD)	64.41 (11.40)	64.17 (11.42)	65.40 (11.28)	0.087
OCSP, *n* (%)				
PACI	895 (56.4)	728 (57.2)	167 (53.0)	0.177
TACI	83 (5.2)	62 (4.9)	21 (6.7)	0.201
LACI	104 (6.6)	81 (6.4)	23 (7.3)	0.549
POCI	505 (31.8)	401 (31.5)	104 (33.0)	0.006
Stroke severity				
Mild	1093 (68.9)	889 (69.9)	204 (64.8)	<0.001
Moderate	367 (23.1)	289 (22.7)	78 (24.8)	0.442
Severe	127 (8.0)	94 (7.4)	33 (10.5)	0.071
Neurological function^a^				
NIHSS	5 (7)	5 (7)	6 (7)	0.006
BI	60 (50)	65 (50)	55 (50)	0.004
mRS	3 (2)	3 (2)	3 (2)	0.007
Risk factors, *n* (%)				
Hypertension	1162 (73.2)	908 (71.4)	254 (80.6)	0.001
Diabetes	472 (29.7)	366 (28.8)	106 (33.7)	0.090
Atrial fibrillation	71 (4.5)	52 (4.1)	19 (6.0)	0.135
Artery stenosis	416 (26.2)	341 (26.8)	75 (23.8)	0.279
Obesity	157 (9.9)	100 (7.9)	57 (18.1)	<0.001
Current smoking	669 (42.2)	628 (49.4)	41 (13.0)	<0.001
Alcohol drinking	339 (21.4)	335 (26.3)	4 (1.3)	<0.001

*OCSP* Oxfordshire Community Stroke Project, *TACI* total anterior circulation infarct, *PACI* partial anterior circulation infarct, *POCI* posterior circulation infarct, *LACI* lacunar infarct, *NIHSS* National Institute of Health stroke scale, *BI* Barthel index, *mRS* modified Rankin scale

^a^Data were presented as median with interquartile range

### Sex differences in outcomes among patients with atherothrombotic infarction and low TC levels

Table 2 shows that women had significantly higher recurrence and dependency rates at 12 months than men did; the corresponding rates were 33.2 vs. 25.1% (*P* = 0.010) for recurrence rate at 12 months and 31.3 vs. 24.5% (*P* = 0.031) for dependency rate at 12 months. However, there were no significant differences in mortality at 3 and 12 months after stroke or in recurrence and dependency rates at 3 months after stroke.

**Table 2:** Sex differences in outcomes among atherosclerotic stroke patients with low TC level

Outcomes	Men	Women	*P*
At 3 months after stroke			
Mortality, *n* (%)	63 (6.3)	13 (5.7)	0.736
Recurrence, *n* (%)	94 (10.0)	24 (11.1)	0.630
Dependency, *n* (%)	99 (10.5)	25 (11.6)	0.659
At 12 months after stroke			
Mortality, *n* (%)	95 (8.8)	23 (8.6)	0.945
Recurrence, *n* (%)	253 (25.1)	83 (33.2)	0.010
Dependency, *n* (%)	242 (24.5)	76 (31.3)	0.031

### Sex differences in risk factors for outcomes at 3 and 12 months after stroke among patients with atherothrombotic infarction and low TC levels

Results of the univariate analysis indicated that age and stroke severity were significantly associated with outcomes at 3 and 12 months after stroke. Moreover, OCSP classification, diabetes mellitus (DM), arterial stenosis, and alcohol drinking were associated with mortality, and sex, obesity, and current smoking were associated with recurrence and dependency rates (Table 3).

**Table 3:** Unadjusted OR with 95% CI of outcome determinants at 3 and 12 months after stroke among patients with low TC level in univariate analysis

Factors	Reference	Mortality		Recurrence		Dependency	
		3 months	12 months	3 months	12 months	3 months	12 months
Men	Women	0.94 (0.56, 1.60)	0.98 (0.61, 1.58)	1.02 (0.66, 1.57)	1.48 (1.10, 2.00)*	1.10 (0.73, 1.66)	1.40 (1.03, 1.91)*
Age	–	1.07 (1.05, 1.10)*	1.07 (1.05, 1.10)*	1.03 (1.01, 1.05)*	1.02 (1.01, 1.04)*	1.03 (1.02, 1.05)*	1.02 (1.01, 1.04)*
OCSP	POCI						
PACI		0.73 (0.47, 1.15)	0.94 (0.62, 1.44)	0.97 (0.66, 1.42)	1.03 (0.78, 1.36)	1.00 (0.69, 1.46)	1.05 (0.79, 1.39)
TACI		1.91 (0.90, 4.02)	2.46 (1.21, 5.02)*	1.72 (0.84, 3.51)	1.70 (0.93, 3.09)	1.86 (0.93, 3.72)	1.65 (0.89, 3.08)
LACI		0.25 (0.06, 1.06)	0.44 (0.15, 1.25)	0.56 (0.23, 1.35)	0.74 (0.43, 1.27)	0.65 (0.28, 1.47)	0.70 (0.40, 1.22)
Stroke severity	Mild						
Moderate		5.02 (2.82, 8.93)*	3.80 (2.31, 6.26)*	1.88 (1.27, 2.77)*	2.32 (1.74, 3.08)*	1.85 (1.26, 2.72)*	2.31 (1.73, 3.09)*
Severe		32.18 (18.02, 57.47)*	32.44 (18.99, 55.43)*	3.74 (2.11, 6.66)*	2.44 (1.39, 4.30)*	4.43 (2.55, 7.67)*	2.15 (1.16, 4.00)*
Hypertension	No	0.71 (0.46, 1.11)	0.73 (0.49, 1.08)	1.08 (0.73, 1.61)	1.24 (0.93, 1.64)	1.16 (0.78, 1.72)	1.26 (0.94, 1.69)
Diabetes	No	0.57 (0.34, 0.95)^*^	0.56 (0.35, 0.90)*	0.95 (0.65, 1.39)	1.12 (0.85, 1.47)	0.89 (0.61, 1.29)	1.20 (0.91, 1.57)
AF	No	1.24 (0.49, 3.17)	1.66 (0.73, 3.77)	0.63 (0.22, 1.75)	1.05 (0.55, 2.02)	0.59 (0.21, 1.65)	0.93 (0.46, 1.85)
Artery stenosis	No	0.50 (0.29, 0.88)*	0.59 (0.36, 0.97)*	1.14 (0.78, 1.66)	1.08 (0.81, 1.42)	1.10 (0.75, 1.59)	1.09 (0.82, 1.45)
Obesity	No	0.50 (0.20, 1.25)	0.45 (0.18, 1.12)	0.90 (0.49, 1.63)	1.84 (1.23, 2.76)*	0.85 (0.47, 1.54)	1.94 (1.29, 2.91)*
Current smoking	No	0.79 (0.52, 1.22)	0.85 (0.58, 1.26)	0.71 (0.50, 1.02)	0.74 (0.57, 0.95)*	0.68 (0.47, 0.96)*	0.72 (0.56, 0.94)*
Alcohol drinking	No	0.57 (0.32, 1.04)	0.55 (0.32, 0.94)*	0.67 (0.42, 1.07)	0.84 (0.62, 1.14)	0.60 (0.37, 0.96)*	0.84 (0.62, 1.15)

*OR* odds ratios, *CI* confidence intervals; ^*^Presented *P*<0.05 in univariate analysis

The sex differences in recurrence and dependency rates became non-significant after adjustment for age, severity, subtype, and risk factors. Older age and stroke severity were independent risk factors for stroke outcomes. A low risk of mortality was observed in patients with DM at 12 months after stroke, but a positive association was found between obesity and the recurrence and dependency rates at 12 months after stroke. The risk of mortality decreased by 49% at 12 months in patients with DM (*P* = 0.013). However, the risk increased by 77% for recurrence (*P* = 0.008) and by 87% for dependency (*P* = 0.004) at 12 months in patients with obesity (Table 4).

**Table 4:** Adjusted OR with 95% CI of outcome determinants at 3 and 12 months after stroke among patients with low TC level in multivariate analysis

Factors	Reference	Mortality		Recurrence		Dependency	
		3 months	12 months	3 months	12 months	3 months	12 months
Men	Women	–	–	–	1.27 (0.92, 1.75)	–	1.18 (0.84, 1.64)
Age	–	1.06 (1.03, 1.08)*	1.06 (1.04, 1.08)*	1.03 (1.01, 1.04)*	1.02 (1.01, 1.03)*	1.03 (1.01, 1.04)*	1.02 (1.01, 1.03)*
OCSP	POCI						
PACI		–	0.77 (0.47, 1.25)	–	–	–	–
TACI		–	0.80 (0.33, 1.99)	–	–	–	–
LACI		–	0.59 (0.19, 1.86)	–	–	–	–
Stroke severity	Mild						
Moderate		4.86 (2.72, 8.70)*	3.77 (2.25, 6.30)*	1.86 (1.26, 2.75)*	2.27 (1.70, 3.03)*	1.84 (1.24, 2.71)*	2.28 (1.70, 3.05)*
Severe		26.23 (14.47, 47.55)*	27.46 (15.39, 49.02)*	3.39 (1.89, 6.06)*	2.27 (1.28, 4.02)*	3.92 (2.24, 6.85)*	1.96 (1.05, 3.68)*
Diabetes	No	–	0.51 (0.30, 0.87)*	–	–	–	–
Artery stenosis	No	0.68 (0.37, 1.24)	0.73 (0.42, 1.28)	–	–	–	–
Obesity	No	–	–	–	1.77 (1.17, 2.70)*	–	1.87 (1.23, 2.84)*
Current smoking	No	–	–	–	0.92 (0.70, 1.22)	0.90 (0.60, 1.35)	0.89 (0.67, 1.19)
Alcohol drinking	No	–	0.47 (0.42, 1.49)	–	–	0.76 (0.45, 1.30)	–

*OR* odds ratios, *CI* confidence intervals; ^*^Presented *P*<0.05 after adjusted by covariates

## Discussion

This is the first report to demonstrate sex differences in clinical features, risk factors, and outcomes among patients with atherothrombotic infarction and low TC levels. Women were more likely than men to have PACI, poor neurological function, hypertension, and obesity, but men were more likely than women to be current smokers or to drink alcohol. There were significantly higher recurrence and dependency rates at 12 months in women than in men. However, the sex differences in stroke outcomes disappeared after adjustment for age, stroke severity, subtype, and risk factors.

Previous studies have indicated that women tend to have strokes at an older age than men do [22–24]. It has been reported that women were more likely than men to have severe stroke [23, 25], but this result was not found in other studies [26, 27]. Moreover, a greater prevalence of hypertension, DM, atrial fibrillation, dyslipidemia, and obesity has been reported for women in previous studies [20, 28, 29]. Consistent with these studies, in the present study, we found that women were more likely than men to have POCI, severe stroke, hypertension, and obesity but less likely to be current smokers and to consume alcohol. The delayed time to hospital admission in women could explain the greater frequency of severe stroke [30].

High cholesterol level is an identified risk factor for coronary heart disease, but its role in stroke remains controversial. The associations between high serum TC levels and an increased risk of ischemic stroke have been reported in several studies [31, 32], but a clear association was not found in others [33–37]. A positive association between TC levels and atherothrombotic infarction has been reported in previous studies [38, 39]. Other studies have indicated that a higher TC level increased the risk of cerebral infarction [9, 40], but sex differences in the association between TC level and stroke outcomes are not well-known.

TC levels were associated with increased risk of severe stroke, TACI, and poor functional outcomes in patients with ischemic stroke who had received pre-stroke statin treatment, and the short-term and long-term mortality rates were significantly higher in patients with low cholesterol levels [41].

Poor outcomes after AIS have been reported in patients with low cholesterol levels [9, 40, 41]. In particular, a negative or non-significant association between TC level and mortality was observed in patients with ischemic stroke aged 70 years or older [35]. Higher TC levels were also associated with lower short-term mortality after stroke; the neuroprotective role of cholesterol may have contributed to this finding [11, 42, 43]. Another study indicated that the lower mortality after stroke among patients with higher cholesterol levels attributed to hypercholesterolemia could be linked to minor strokes (mainly small-vessel stroke) with good outcomes [44]. Several large-scale studies demonstrated that lower TC levels were associated with higher all-cause mortality and that higher TC levels were associated with lower all-cause mortality [45, 46]. Furthermore, a U-shaped association between TC level and dependency after AIS was shown in a group of people of very old age; patients with moderate TC levels had the most favorable outcomes after AIS in patients aged >80 years [47]. Moreover, our previous study indicated that low cholesterol levels among patients with atherothrombotic infarction receiving statin treatment increased long-term dependency and recurrence rates, but not mortality rates [48].

Consistent with the results of previous studies, in the present study, higher recurrence and dependency rates at 12 months after stroke were observed for women than for men. The sex differences in recurrence and dependency rates became statistically non-significant after adjustment for covariates. Moreover, older age and stroke severity were independent risk factors for stroke outcomes in this study. A low risk of mortality was observed in patients with DM at 12 months after stroke, but a positive association was found between obesity and recurrence and dependency rates at 12 months after stroke. The negative association between DM and mortality at 12 months after stroke could be explained by treating patients with metformin, which mediates enhanced angiogenesis [49].

Statin therapy has become the most important advancement in stroke prevention since aspirin and blood pressure-lowering therapies were introduced. Clinical trials have shown that lowering cholesterol levels can reduce the incidence of stroke in high-risk populations and in patients with a stroke or transient ischemic attack [37, 50]. However, in this study, worse long-term dependency and recurrence rates occurred in female patients with low TC levels on admission, and all patients received statin treatment after stroke. Thus, the benefit of statins for improving outcomes after stroke in Asian populations, especially in Chinese people, needs to be explored further.

There are several limitations in this study. First, all patients were from a local neurological hospital in Tianjin, China, and may not represent all stroke patients in China. Second, data on statin use before stroke onset were lacking, which may have affected the evaluation of TC level on stroke outcomes. However, the aim of this study was to evaluate the differences in stroke outcomes between men and women with low TC levels. Thus, it is not likely that the lack of information regarding previous statin use had a major impact on the results. Third, there were a few patients who completed follow-up by telephone (2.8% at 3 months and 12.2% at 12 months), which may have introduced an assessment bias due to a measurement disparity. Moreover, the differences in baseline characteristics (higher prevalence of DM at 3 months; older age, higher frequency of severe stroke, and higher prevalence of DM at 12 months) may have partially affected the assessment of the association between outcomes and risk factors. Finally, the TC compositions were not measured in this study, which may have affected the evaluation of stroke outcomes among patients with a low TC level.

## Conclusions

This large, hospital-based, prospective study was the first to report sex differences in outcomes at 3 and 12 months after stroke among patients with atherothrombotic infarction and low TC levels. Women were more likely than men to have POCI, severe stroke, hypertension, and obesity and were less likely to smoke or consume alcohol. There were no sex differences in stroke outcomes. Older age and severe stroke were common risk factors for poor outcomes after stroke in this study. DM was an independent predictor of low mortality at 12 months after stroke, which could be explained by metformin’s mediation of enhancing angiogenesis. Obesity was the determinant of recurrence and dependency rates at 12 months after stroke. These findings suggest that patients (both men and women) with atherothrombotic infarction and low TC levels would not benefit from receiving statin treatment. Therefore, it is crucial to explore the impact of statin treatment on outcomes in Asian patients, especially Chinese atherothrombotic infarction patients, with low TC levels in order to improve outcomes after stroke and reduce the disease burden.
